# Influence of Dose and Extraction Method of Biostimulants on Drought Stress Tolerance in *Coleus amboinicus* Lour. Plants

**DOI:** 10.3390/plants15142107

**Published:** 2026-07-08

**Authors:** Fabio Scotto Di Covella, Luca Leotta, Agnese Carchiolo, Daniela Romano, Marta Fibiani, Antonella Calzone, Antonio Ferrante, Stefania Toscano

**Affiliations:** 1 Institute of Crop Science, Sant’Anna School of Advanced Studies of Pisa, 56127 Pisa, Italy; fabio.scottodicovella@santannapisa.it (F.S.D.C.); antonio.ferrante@santannapisa.it (A.F.); 2 Department of Agriculture, Food and Environment (Di3A), Università degli Studi di Catania, Via Santa Sofia 100, 95123 Catania, Italy; luca.leotta@phd.unict.it (L.L.); agnese.carchiolo@pdh.unict.it (A.C.); 3 CREA Research Centre for Engineering and Agro-Food Processing, via G. Venezian 26, 20133 Milano, Italy; marta.fibiani@crea.gov.it (M.F.); antonella.calzone@crea.gov.it (A.C.); 4 Department of Veterinary Sciences, Università degli Studi di Messina, Viale G. Palatucci s.n., 98168 Messina, Italy; stefania.toscano@unime.it

**Keywords:** abiotic stress, chlorophyll *a* fluorescence, gas exchange, ornamental plants

## Abstract

This study addresses the challenge of drought stress in ornamental plants by evaluating the drought response of Cuban oregano (*Coleus amboinicus* Lour.) and the potential of sea fennel (*Crithmum maritimum* L.) extracts as biostimulants to mitigate the effects of water deficit. Conducted in a greenhouse under two irrigation regimes, full irrigation (100% water replacement) and deficit irrigation (50%), the research applied foliar treatments of sea fennel extracts prepared via aqueous extraction (WE) and alcoholic extraction (AE) at various concentrations. Drought stress significantly reduced total dry biomass by 48%, leaf number by 33%, and leaf area by 57%, severely impacting both aboveground and root growth. Biostimulant treatments alleviated these negative effects, with alcoholic extracts at 2.5 mL L^−1^ showing the greatest efficacy by increasing biomass by approximately 60% and restoring leaf area to levels comparable to fully irrigated plants. Aqueous extracts provided moderate improvements. Drought induced an increased root-to-shoot ratio, indicating adaptive biomass allocation, while SPAD values and gas exchange parameters (net photosynthesis and stomatal conductance) declined under stress but improved with biostimulant application, especially with AE treatments. Photosystem II efficiency (Fv/Fm) confirmed stress but showed partial recovery in AE-treated plants. Additionally, WE at 5 mL L^−1^ enhanced anthocyanin and amino acid accumulation, suggesting metabolic adjustments to drought. Overall, alcoholic sea fennel extracts demonstrated superior potential as sustainable biostimulants to enhance drought tolerance in ornamental plants.

## 1. Introduction

Climate change is anticipated to increase both the frequency and severity of extreme drought events, thereby reducing water availability for plant growth [1]. As a result, the ornamental plant sector faces a significant challenge, since global warming is likely to impact not only global production [2] but also the use of ornamental species in green spaces due to increasingly scarce water resources. Consequently, ornamental horticulture must adapt to new climatic conditions, making global warming a formidable challenge for the sector [3,4].

One potential strategy to alleviate water scarcity is to identify genotypes that demonstrate greater drought tolerance, lower water requirements, or enhanced adaptation to extreme weather conditions [5,6]. Consequently, native species and naturally drought-tolerant plants are crucial in the development of ‘dry’ or xeriscape gardens [7]. A complementary approach involves examining the physiological and morphological mechanisms that underpin plant responses to water stress. Despite extensive research, these mechanisms remain not fully understood [8].

Plants display a diverse array of morphological and physiological adaptations to drought stress [9,10], which are essential for selecting varieties tolerant to water scarcity [11]. Drought stress frequently causes noticeable morphological changes in above-ground tissues, such as smaller leaf size and the abscission of older leaves, which help to reduce transpiration and water loss [12]. While the reduction in leaf area enhances the plant’s water status, it also limits photosynthesis and carbon assimilation.

Roots play a central role in the response and adaptation to drought [13]. The root-to-shoot ratio is commonly used to evaluate biomass allocation [14] and to assess the distribution of photosynthates between above- and below-ground organs [15]. An increase in this ratio indicates a shift in biomass allocation towards underground structures. This strategy enables plants to explore a larger volume of soil to enhance water uptake. According to the “optimal allocation theory” [16], plants preferentially allocate biomass and non-structural carbohydrates to acquire the most limiting resources [17]. For example, drought has been shown to increase the root-to-shoot ratio in rice [18] and sagebrush [6], whereas waterlogging reduces this ratio in winter wheat [19] and maize [20].

Photosynthetic pigments are crucial for the absorption, transfer, and conversion of light energy during photosynthesis [21,22]. Consequently, chlorophyll concentration serves as a reliable indicator of photosynthetic activity [23]. Under abiotic stress, a reduction in chlorophyll content functions as a non-stomatal limitation and acts as a protective mechanism for photosynthetic structures [24,25,26]. Since chlorophyll content directly affects photosynthesis and plant growth [27], drought-induced imbalances between light capture and utilisation can significantly inhibit photosynthetic activity [28].

To minimise water loss during drought stress, stomata may close partially or completely, leading to altered gas exchange in the leaves. Changes in gas exchange are typically assessed using parameters such as stomatal conductance (gs), transpiration rate (E), and relative water content (RWC) [29]. In drought-tolerant plants, RWC decreases progressively as soil moisture declines [30], and stomatal closure consequently reduces photosynthetic activity.

Water use efficiency (WUE), defined as the ratio of photosynthesis to transpiration (Pn/E), is a crucial indicator of a plant’s adaptation to water stress. An increase in WUE under drought conditions is generally linked to tolerance to deficit irrigation, whereas a decrease is typical of more sensitive species [31,32]. However, plants with low WUE may remain more competitive in arid environments by rapidly exploiting available resources, thereby limiting competition. Conversely, species with high WUE usually perform better in low-competition environments, regardless of water availability, likely due to superior water and nitrogen reserves [33]. Therefore, WUE responses to drought vary depending on the genotype and the intensity of the stress [34].

Photosynthesis, a fundamental process for plant growth, is particularly sensitive to drought stress, resulting in significant reductions in carbon assimilation [35]. The physiological status of plants can be assessed by examining the integrity and efficiency of their photosynthetic apparatus [36]. Adverse environmental conditions, such as water stress, can impair the functionality of photosystems. Chlorophyll *a* fluorescence offers an indirect and non-invasive method to evaluate this damage, as fluorescence values increase when the efficiency of photosystem II (PSII) declines due to an imbalance between electron supply and utilisation [37]. The Fv/Fm ratio, which represents the maximum quantum yield of PSII, is widely used as an indicator of plant stress; values between 0.78 and 0.85 indicate non-stressed conditions [36]. Such physiological indicators are essential for understanding drought responses, developing mitigation strategies, and selecting species adapted to water scarcity [38].

In addition to selecting appropriate genotypes, the use of biostimulants is a promising strategy for enhancing drought tolerance. Biostimulants can enhance plant resilience to abiotic stress, increase nutrient use efficiency, and stimulate specialised plant metabolism, thereby improving stress tolerance by regulating metabolic pathways, nutrient uptake, and antioxidant defence systems. They contribute to plant health and productivity across various growth stages [39]. Organic farming systems, in particular, benefit from the application of biostimulants, as these products can alleviate nutrient limitations and help narrow the performance gap between organic and conventional methods [40]. Overall, biostimulants make a significant contribution to agricultural sustainability by improving both the quantity and quality of yields while minimising environmental impacts [41].

There is growing interest in plant-derived biostimulants (PDBs), which are natural products extracted from higher plants and rich in specialised metabolites that can activate physiological responses [42,43]. These extracts are typically rich in secondary metabolites, including polyphenols, flavonoids, vitamins, and other antioxidants, as well as peptides and amino acids, which are known to function as signalling molecules or metabolic regulators. Among these, plant extracts (PEs) obtained through the simple maceration of various plant organs—such as seeds, roots, stems, leaves, bark, and flowers—are of particular interest. Medicinal plants rich in secondary metabolites are frequently used, and recent studies have assessed extracts from liquorice root, lemongrass, garlic, and especially moringa leaves for their biostimulant potential [43]. Despite the wide variety of candidate species, many remain underexplored.

Sea fennel (*Crithmum maritimum* L.) is a promising candidate for this purpose. This halophytic species, belonging to the *Apiaceae* family, is native to Mediterranean Europe and is commonly found along the Italian coastline. Its ability to tolerate high salinity enables it to thrive in coastal environments, often growing in rocky crevices within the intertidal zone. Sea fennel is a rhizomatous perennial characterised by stout, branched stems that are woody at the base and reach heights of 30–60 cm. The species has long been valued in folk medicine for its diuretic, antiscorbutic, digestive, and purgative properties [44]. Several studies have documented the antioxidant and antibacterial properties of its essential oil [45,46,47], while falcarindiol extracted from its leaves has demonstrated strong antibacterial and cytotoxic activity [48]. Its biochemical profile, rich in antioxidants and metabolites related to stress, suggests a potential role in enhancing plant tolerance to drought; however, its effects on plant physiology and stress-response mechanisms have not been fully elucidated.

*Coleus amboinicus* Lour. (syn. *Plectranthus amboinicus* (Lour.) Spreng.) is a perennial herbaceous species belonging to the *Lamiaceae* family, widely distributed across the tropical and warm regions of Africa, Asia, and Australia. In Italy, it is primarily cultivated as an ornamental plant and is commonly referred to as Cuban oregano due to its strong aroma. The species is extensively used in traditional medicine, with numerous phytochemicals identified, including phenols, terpenoids, phenolic acids, flavonoids, flavones, and tannins [49]. Crude extracts of *C. amboinicus* have demonstrated multiple pharmacological activities both in vitro and in vivo [50], and several *Coleus* species are noted for their cytotoxic and anticancer properties [51].

In this study, we examined the response of *C. amboinicus*, a highly ornamental species with moderate drought tolerance, to two irrigation regimes: a well-watered control and a drought-stressed treatment receiving 50% of the water lost through evapotranspiration. Additionally, plants were treated with biostimulants derived from *C. maritimum*, prepared using different extraction methods (aqueous maceration or alcoholic extraction) and concentrations.

This study, representing the first instance of a *C. maritimum* extract being applied to another species, aimed to:

- Evaluate whether a halophytic and ruderal plant, such as *C. maritimum*, and therefore inherently tolerant to adverse conditions, could be used to produce biostimulant products.
- Determine whether bioactive compounds obtained by alcohol extraction are more effective than those obtained by aqueous extraction for counteracting drought stress in *C. amboinicus*.
- Define the optimal dose for the two different extraction methods.
- Verify the efficacy of these products on a species, *C. amboinicus*, grown under drought stress.

## 2. Results

### 2.1. Biomass and Leaf Characteristics

Water deficit significantly affected plant biomass accumulation (Table 1). Plants grown at 50% water container capacity (50% WC) exhibited a marked reduction in total dry biomass compared with the control (100% WC), with a decrease of 48%. Similar reductions were observed for epigeous biomass, leaf dry biomass, stem dry biomass, and root dry biomass (Table 1).

The application of *C. maritimum* extracts partially mitigated the adverse effects of water deficit on plant growth, with responses varying according to both the extraction method and the applied dose. Among the alcoholic extract (AE) treatments, AE at 2.5 mL L^−1^ was the most effective, increasing total dry biomass by approximately 60% compared to untreated plants grown at 50% water capacity (50% WC). This treatment also significantly enhanced individual biomass components, including epigeous dry biomass by about 63%, leaf dry biomass by 52%, stem dry biomass by 75%, and root dry biomass by 53%. For several parameters, biomass values were comparable to those recorded in fully irrigated (100% WC) control plants (Table 1).

Water extracts (WEs) also promoted plant growth under drought conditions, although their effects were less pronounced than those observed with AE treatments. The highest WE dose (10 mL L^−1^) increased total dry biomass by approximately 31% compared to the untreated 50% WC treatment, but this was still lower than both the 100% WC control and the AE 2.5 mL L^−1^ treatment.

As expected, the root-to-shoot ratio increased under water deficit, rising from 0.16 in control plants to 0.22 in 50% WC plants (Table 1), indicating a greater allocation of biomass to root development.

Water deficit also significantly affected leaf morphological traits (Table 2). Plants growing at 50% WC exhibited a notable reduction in both leaf number and total leaf area compared with control plants grown at 100% WC. Specifically, leaf number decreased by approximately 33%, while total leaf area was reduced by 57%. Both unit leaf area and specific leaf area (SLA) were also significantly diminished under drought conditions.

The application of AE treatments effectively alleviated the adverse effects on leaf development. Notably, AE at 2.5 mL L^−1^ yielded the most significant improvement, increasing leaf number by 24% and total leaf area by 132%, with values comparable to those of the control plants. This treatment also resulted in the highest unit leaf area and specific leaf area (SLA) among the drought-stressed plants (Table 2).

WE treatments resulted in moderate improvements in leaf traits under water stress. The 5 mL L^−1^ WE treatment increased total leaf area by 75% compared with untreated plants at 50% WC; however, this value remained lower than that achieved with AE at 2.5 mL L^−1^ (Table 2).

### 2.2. SPAD Index, Gas Exchange, and Chlorophyll a Fluorescence

**Figure 1:**
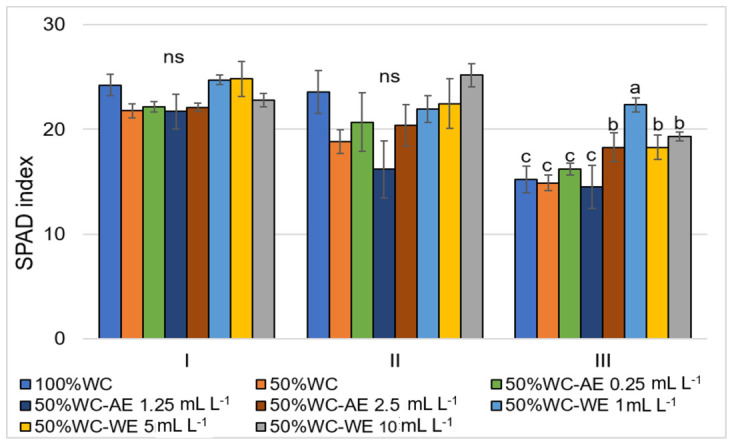
SPAD index values during the representative measurements of the experimental trials—100% WC = received 100% of water considering the evapotranspiration rate performed using the gravimetric method and were foliar spraying to runoff with distilled water; 50% WC = received 50% of water considering the evapotranspiration rate performed using the gravimetric method and were foliar spraying to runoff with distilled water; AE = biostimulant extraction from leaves of *C. maritimum* by ethanol (96%) and methanol (4%); WE = biostimulant obtained from water maceration of leaves of *C. maritimum*. I, II, and III indicate the physiological measurement campaigns performed respectively at 12, 32, and 52 days from the start of the experimental trial. Values are the means ± standard error (SE). Different letters: statistically significantly different means for *p* < 0.05 (Tukey’s test) for each measurement.

SPAD values were generally low and gradually decreased throughout the experimental period, with the highest values recorded during the first measurements (I) and the lowest during the final measurements (III) (Figure 1). No significant differences were observed among treatments during the initial measurements (I).

In the second measurement (II), lower SPAD values were observed across all treatments, although the differences were not statistically significant.

By the final measurements (III), SPAD values had further declined across all treatments. However, higher SPAD index values were observed in plants treated with WE at 1 mL L^−1^ (22 SPAD units), followed by AE at 2.5 mL L^−1^, WE at 5 mL L^−1^, and AE at 10 mL L^−1^.

**Figure 2:**
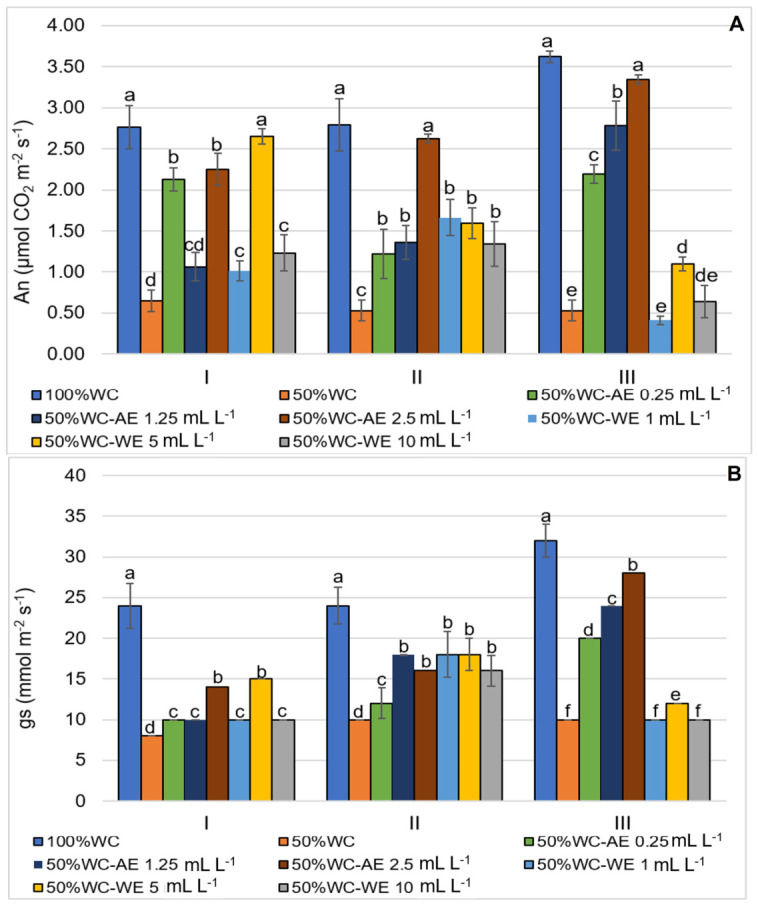
Net photosynthesis (A_n_) (**A**) and stomatal conductance (g_s_) (**B**) during the representative measurements of the experimental trials—100% WC = received 100% of water considering the evapotranspiration rate performed using the gravimetric method and were foliar spraying to runoff with distilled water; 50% WC = received 50% of water considering the evapotranspiration rate performed using the gravimetric method and were foliar spraying to runoff with distilled water; AE = biostimulant extraction from leaves of *C. maritimum* by ethanol (96%) and methanol (4%); WE = biostimulant obtained from water maceration of leaves of *C. maritimum*. I, II, and III indicate the physiological measurement campaigns performed respectively at 12, 32, and 52 days from the start of the experimental trial. Values are the means ± standard error (SE). Different letters: statistically significantly different means for *p* < 0.05 (Tukey’s test) for each measurement.

The net photosynthetic rate (A_n_) varied significantly between treatments and over the sampling periods (Figure 2A). In the first survey (I), the highest A_n_ values were recorded in well-watered plants (100% WC) and in those treated with WE at 5 mL L^−1^, while lower values were observed in plants subjected to water deficit (50% WC) and in those treated with AE at 1.25 mL L^−1^.

In the second set of measurements (II), A_n_ remained high in plants at 100% WC and in those treated with AE at 2.5 mL L^−1^, whereas the lowest values were once again observed in the 50% WC treatment.

By the final measurements (III), A_n_ showed a slight decline in most treatments; however, relatively higher values were maintained in control plants (100% WC) and in plants treated with AE at all tested doses (0.25, 1.25, and 2.5 mL L^−1^). Stomatal conductance (g_s_) exhibited a similar pattern across treatments and sampling times (Figure 2B).

**Figure 3:**
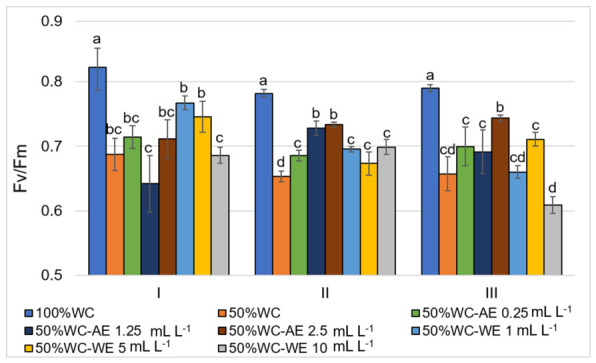
Maximum quantum efficiency of PSII (Fv/Fm) during the representative measurements of the experimental trials—100% WC = received 100% of water considering the evapotranspiration rate performed using the gravimetric method and were foliar spraying to runoff with distilled water; 50% WC = received 50% of water considering the evapotranspiration rate performed using the gravimetric method and were foliar spraying to runoff with distilled water; AE = biostimulant extraction from leaves of *C. maritimum* by ethanol (96%) and methanol (4%); WE = biostimulant obtained from water maceration of leaves of *C. maritimum*. I, II, and III indicate the physiological measurement campaigns performed respectively at 12, 32, and 52 days from the start of the experimental trial. Values are the means ± standard error (SE). Different letters: statistically significantly different means for *p* < 0.05 (Tukey’s test) for each measurement.

The maximum quantum efficiency of PSII (Fv/Fm) generally remained below the stress threshold of 0.82 across all treatments (Figure 3). In the first survey (I), the highest Fv/Fm values were recorded in plants grown under well-watered conditions (100% WC), with no significant differences observed among the other treatments.

In the second measurement (II), Fv/Fm remained relatively high in 100% WC plants and in those treated with AE at 1.25 and 2.5 mL L^−1^, while the lowest values were once again observed under water deficit conditions (50% WC).

By the final measurement (III), higher Fv/Fm values were maintained in control plants (100% WC) and in plants treated with AE at 2.5 mL L^−1^, indicating a partial mitigation of PSII photoinhibition under drought stress.

### 2.3. Chlorophyll and Carotenoid Content

**Figure 4:**
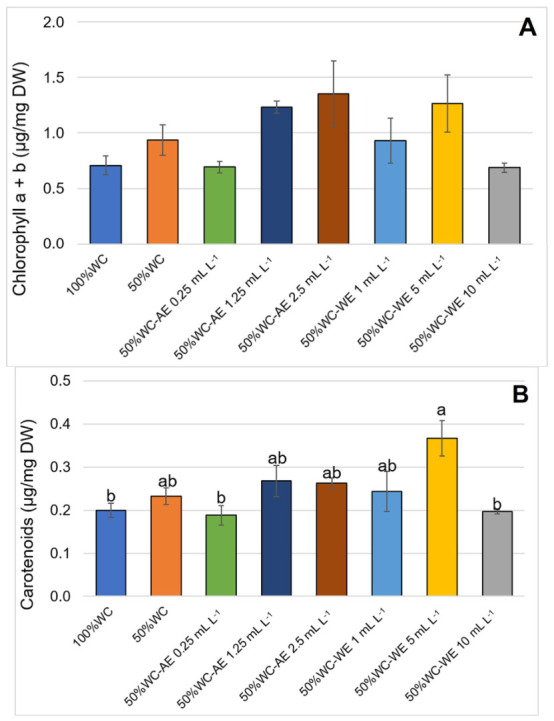
Chlorophyll *a* + *b* (**A**) and carotenoid concentration (**B**) in leaf tissue at the end of the experimental period—100% WC = received 100% of water considering the evapotranspiration rate performed using the gravimetric method and were foliar spraying to runoff with distilled water; 50% WC = received 50% of water considering the evapotranspiration rate performed using the gravimetric method and were foliar spraying to runoff with distilled water; AE = biostimulant extraction from leaves of *C. maritimum* by ethanol (96%) and methanol (4%); WE = biostimulant obtained from water maceration of leaves of *C. maritimum*. Values are expressed on a dry weight basis and represent the mean ± SE. Different letters indicate statistically significant differences (*p* ≤ 0.05).

The spectrophotometric determination of chlorophyll *a* + *b*, although not showing statistically significant differences, revealed a consistent trend towards higher photosynthetic pigment content in plants treated with AE at 1.25 and 2.5 mL L^−1^ and with WE at 5 mL L^−1^, compared with both the control and the 50% WC treatment (Figure 4A). Chlorophyll data were expressed on a dry weight basis to avoid potential misinterpretation associated with fresh weight measurements, which can be misleading under drought stress conditions due to variable tissue water loss.

Carotenoid concentration was significantly higher in WE at the 5 mL L^−1^ treatment (Figure 4B).

### 2.4. Anthocyanin Content

**Figure 5:**
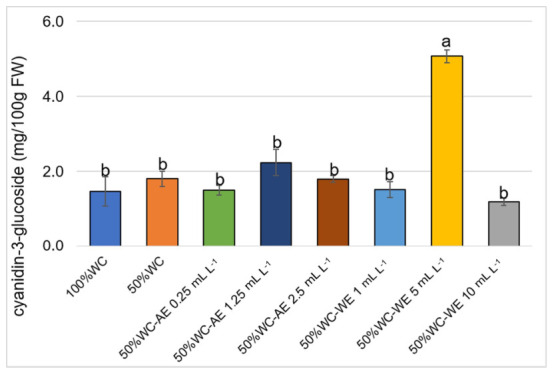
Anthocyanin concentration in leaf tissue at the end of the experimental period expressed as cyanidin-3-glucoside content—100% WC = received 100% of water considering the evapotranspiration rate performed using the gravimetric method and were foliar spraying to runoff with distilled water; 50% WC = received 50% of water considering the evapotranspiration rate performed using the gravimetric method and were foliar spraying to runoff with distilled water; AE = biostimulant extraction from leaves of *C. maritimum* by ethanol (96%) and methanol (4%); WE = biostimulant obtained from water maceration of leaves of *C. maritimum*. Values are expressed on a fresh weight basis and represent the mean ± SE. Different letters indicate statistically significant differences (*p* ≤ 0.05).

In this study, anthocyanins, expressed as cyanidin-3-glucoside equivalents, were significantly higher in the WE 5 mL L^−1^ treatment compared with the other treatments (Figure 5), indicating a strong activation of anthocyanin biosynthesis triggered by the application of the water extract at this concentration.

### 2.5. Nitrate and Reducing Sugar Content

**Figure 6:**
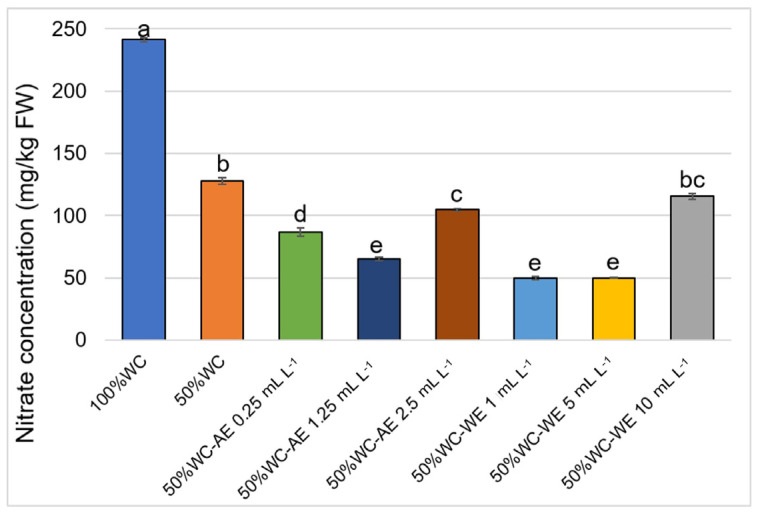
Nitrate concentration in leaf tissue at the end of the experimental trial—100% WC = received 100% of water considering the evapotranspiration rate performed using the gravimetric method and were foliar spraying to runoff with distilled water; 50% WC = received 50% of water considering the evapotranspiration rate performed using the gravimetric method and were foliar spraying to runoff with distilled water; AE = biostimulant extraction from leaves of *C. maritimum* by ethanol (96%) and methanol (4%); WE = biostimulant obtained from water maceration of leaves of *C. maritimum*. Values are expressed on a fresh weight basis and represent the mean ± SE. Different letters indicate statistically significant differences (*p* ≤ 0.05).

Nitrate concentration showed a marked decrease in all treatments compared to fully irrigated conditions (Figure 6). Lower concentrations were observed in 1.25 mL L^−1^ AE, 1 or 5 mL L^−1^ WE.

**Figure 7:**
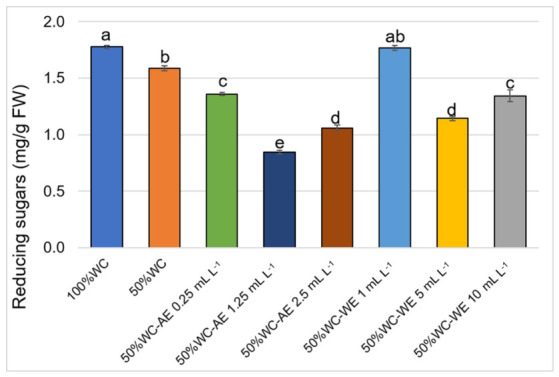
Reducing sugars concentration in leaf tissue at the end of the experimental period—100% WC = received 100% of water considering the evapotranspiration rate performed using the gravimetric method and were foliar spraying to runoff with distilled water; 50% WC = received 50% of water considering the evapotranspiration rate performed using the gravimetric method and were foliar spraying to runoff with distilled water; AE = biostimulant extraction from leaves of *C. maritimum* by ethanol (96%) and methanol (4%); WE = biostimulant obtained from water maceration of leaves of *C. maritimum*. Values are expressed on a fresh weight basis and represent the mean ± SE. Different letters indicate statistically significant differences (*p* ≤ 0.05).

Reducing sugars were significantly lower in all treatments except for the 1 mL L^−1^ WE treatment, which showed values comparable to those of fully irrigated plants. Water stress reduced the levels of reducing sugars, while the treatments mitigated this effect (Figure 7).

### 2.6. Amino Acid Profiles with HPLC-MS/MS Quantification

**Figure 8:**
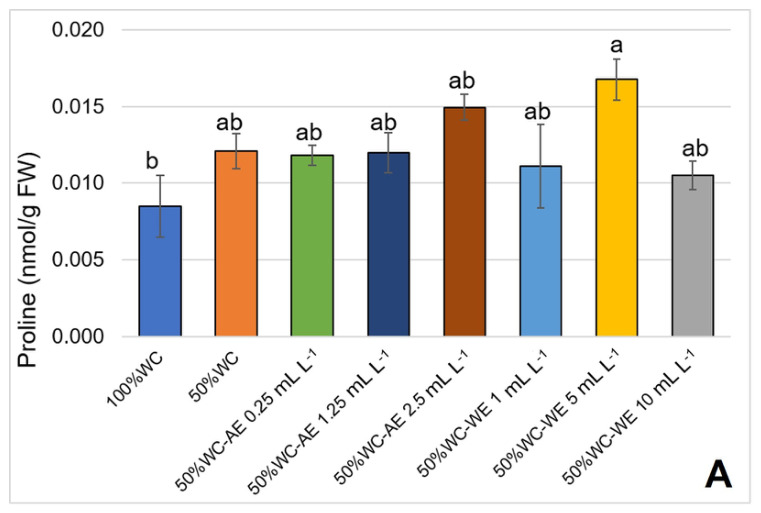

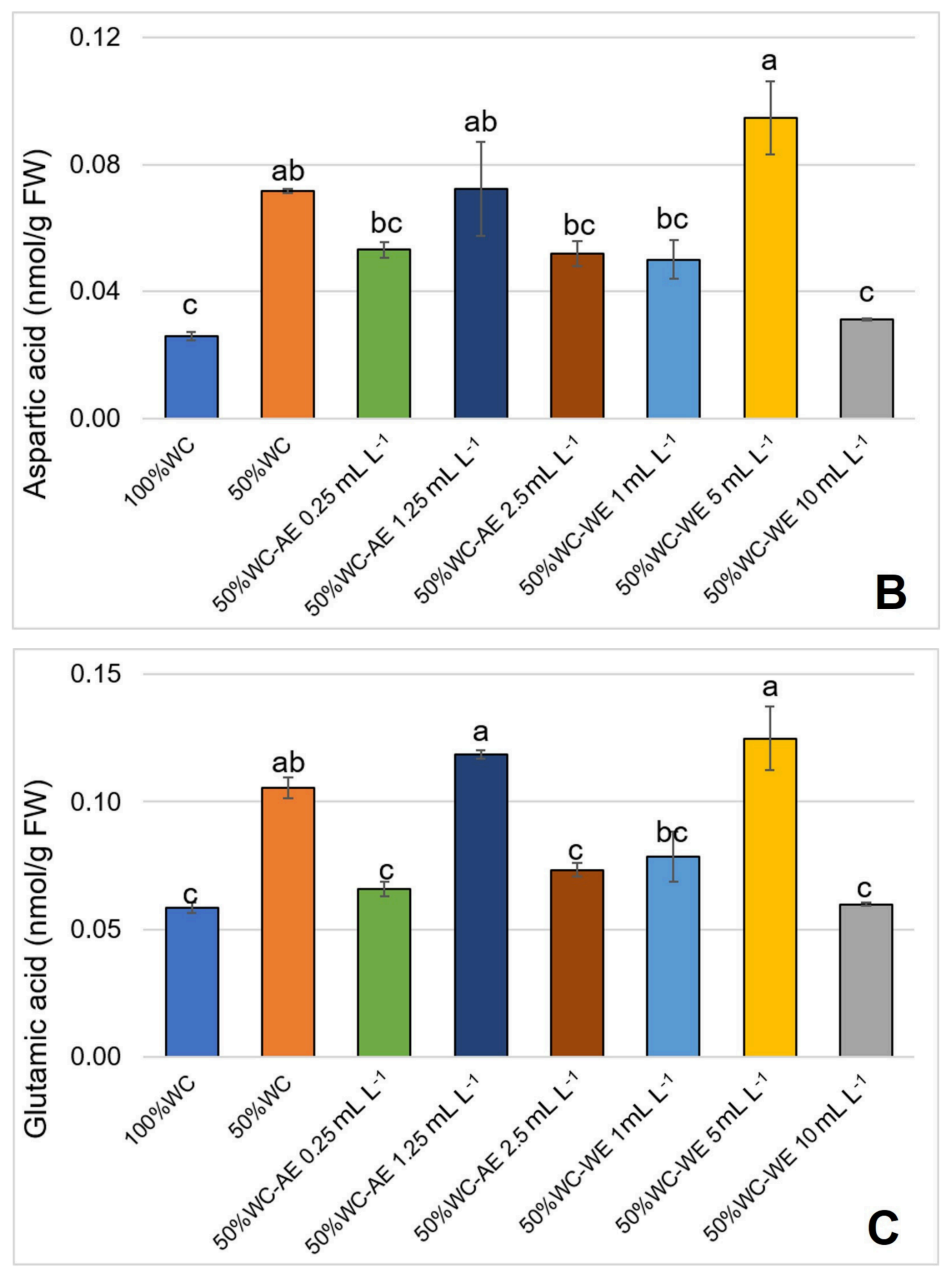
Proline concentration (**A**), aspartic acid concentration (**B**), and glutamic acid concentration (**C**) in leaf tissue at the end of the experimental period—100% WC = received 100% of water considering the evapotranspiration rate performed using the gravimetric method and were foliar spraying to runoff with distilled water; 50% WC = received 50% of water considering the evapotranspiration rate performed using the gravimetric method and were foliar spraying to runoff with distilled water; AE = biostimulant extraction from leaves of *C. maritimum* by ethanol (96%) and methanol (4%); WE = biostimulant obtained from water maceration of leaves of *C. maritimum*. Values are expressed on a fresh weight basis and represent the mean ± SE. Different letters indicate statistically significant differences (*p* ≤ 0.05).

Amino acid analysis using HPLC-MS/MS revealed a significant accumulation of proline in leaf tissue under the WE 5 mL L^−1^ treatment compared to both the other treatments and fully irrigated conditions (Figure 8A).

Aspartic acid also increased significantly under the 5 mL L^−1^ WE treatment (Figure 8B), showing more than a threefold rise compared to the control (100% WC). Similarly, glutamic acid reached its highest levels in the 1.25 mL L^−1^ AE and 5 mL L^−1^ WE treatments (Figure 8C). The quantification of the other amino acid profiles is presented in Table S1.

**Figure 9:**
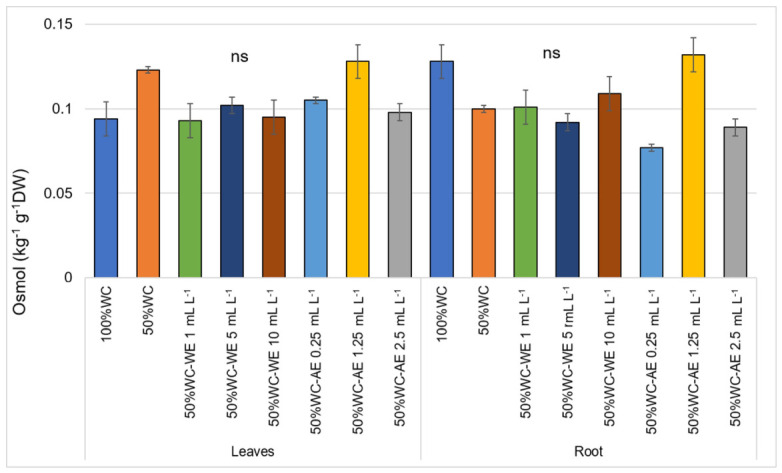
Osmolyte concentration in leaves and roots at the end of the experimental period—100% WC = received 100% of water considering the evapotranspiration rate performed using the gravimetric method and were foliar spraying to runoff with distilled water; 50% WC = received 50% of water considering the evapotranspiration rate performed using the gravimetric method and were foliar spraying to runoff with distilled water; AE = biostimulant extraction from leaves of *C. maritimum* by ethanol (96%) and methanol (4%); WE = biostimulant obtained from water maceration of leaves of *C. maritimum*. Values are expressed on a fresh weight basis and represent the mean ± SE.

Osmolyte concentrations in both leaves and roots did not show a significant difference compared to the control across the various biostimulant doses (Figure 9).

**Figure 10:**
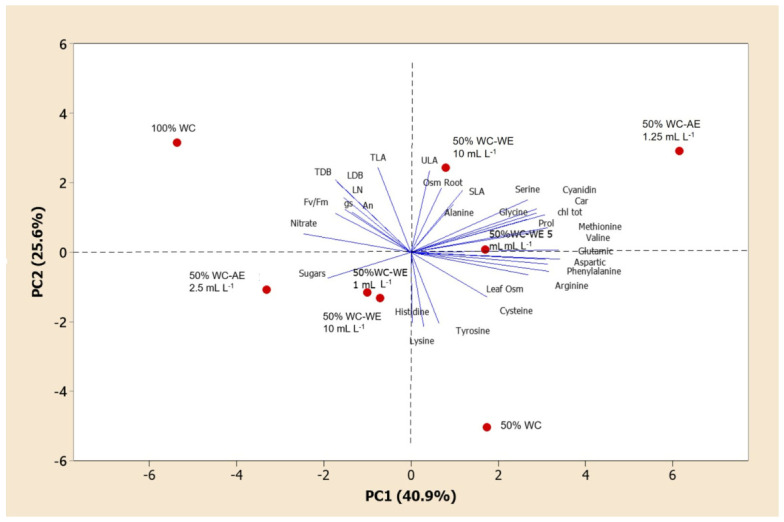
Biplot graph and scores based on the first two components of morphometric, physiological, and biochemical traits at the end of the experimental period of *Coleus amboinicus* response to treatments—100% WC = received 100% of water considering the evapotranspiration rate performed using the gravimetric method and were foliar spraying to runoff with distilled water; 50% WC = received 50% of water considering the evapotranspiration rate performed using the gravimetric method and were foliar spraying to runoff with distilled water; AE = biostimulant extraction from leaves of *C. maritimum* by ethanol (96%) and methanol (4%); WE = biostimulant obtained from water maceration of leaves of *C. maritimum*. Total dry biomass = TDB; leaf dry biomass = LDB; leaf number = LN; total leaf area = TLA; unit leaf area = ULA; specific leaf area = SLA; net photosynthesis = A_n_; stomatal conductance = g_s_; chlorophyll *a* fluorescence = Fv/Fm; leaf osmolite = Leaf Osm; root osmolite = Osm Root; cyanidin-glucoside = Cyanidin; carotenoids = Car; total chlorophyll = Chl tot; proline = Prol; aspartic acid = Aspartic; glutamic acid = Glutamic.

Principal component analysis (PCA) accounted for 66.5% of the total variance, with PC1 and PC2 explaining 40.9% and 25.6% of the variability, respectively (Figure 10). The score plot revealed a clear separation between treatments, indicating distinct physiological and biochemical responses to water availability and biostimulant applications.

PC1 primarily distinguished the treatments based on plant physiological performance and metabolic responses to water deficit. Negative PC1 scores corresponded with higher values of net photosynthetic rate (A_n_), stomatal conductance (g_s_), maximum quantum efficiency of PSII (Fv/Fm), leaf number (LN), leaf dry biomass (LDB), total dry biomass (TDB), and nitrate concentration, indicating superior plant growth and photosynthetic activity. Conversely, positive PC1 scores were linked to the accumulation of amino acids, including arginine, phenylalanine, aspartic acid, glutamic acid, valine, methionine, proline, glycine, and serine, alongside osmotic adjustment traits. Positive PC2 values were associated with total leaf area (TLA), unit leaf area (ULA), specific leaf area (SLA), and root osmotic potential, whereas negative PC2 values were mainly related to histidine, lysine, tyrosine, cysteine, and leaf osmotic traits.

The well-watered control (100% WC), located in the upper-left quadrant, exhibited a strong association with photosynthetic and growth-related variables such as A_n_, g_s_, Fv/Fm, LN, LDB, and TDB, indicating optimal physiological performance under sufficient water supply. In contrast, plants subjected to water deficit (50% WC), positioned in the lower-right quadrant, were linked to the accumulation of several amino acids, suggesting metabolic adjustments in response to drought stress.

Biostimulant treatments under water deficit occupied intermediate positions within the ordination space, indicating a modulation of drought responses. Plants treated with the alcoholic extract (AE) at 1.25 mL L^−1^ under 50% WC were positioned in the upper-right quadrant and showed a positive association with osmotic adjustment and amino acid accumulation. Treatments with the aqueous extract (WE) displayed intermediate distributions, while the AE treatment at 2.5 mL L^−1^ was located closer to the negative side of PC1 and was linked to sugar accumulation.

## 3. Discussion

Water availability in urban and peri-urban Mediterranean areas is becoming increasingly scarce, primarily due to climate change. This decline in water resources adversely impacts the growth, flowering, and visual appeal of herbaceous bedding plants commonly used in urban green spaces [36]. Annual species, frequently planted in flowerbeds, are especially vulnerable, as their cultivation typically demands regular irrigation, rendering them unsuitable in conditions of water scarcity. Consequently, identifying drought-tolerant ornamental species that can preserve their aesthetic quality under water stress is a crucial priority [52].

The selection of such species typically depends on analysing their physiological and biochemical responses to drought stress [5]. However, this process is complicated by both interspecific and intraspecific variability, as drought responses can vary among species within the same genus [53]. At present, insufficient information restricts the rational selection of ornamental plants for use in urban environments.

Our findings indicate that Cuban oregano (*Coleus amboinicus*) employs several drought-avoidance strategies, including altered biomass allocation between roots and shoots, reduced leaf size, and a decrease in total leaf area under drought conditions. These responses align with previous studies reporting reduced biomass production when water availability is limited [54,55]. Notably, the increased root-to-shoot ratio under water deficit reflects a preferential allocation of resources to root development, thereby enhancing water uptake capacity. This response is well documented as a common drought-adaptive strategy [56,57], which reduces water consumption [58] and improves soil water exploration [59]. This shift indicates an adaptive reallocation of resources designed to optimise soil water exploration under stress conditions.

Consistent with earlier findings [60], shoot growth was more markedly inhibited than root growth, suggesting that plants prioritise below-ground development under stress. This was further supported by the observed increase in root biomass, which expanded the absorptive surface area and aided drought avoidance without causing significant reductions in shoot biomass.

Water deficit significantly affected leaf morphology. A reduction in leaf area, caused by limited leaf expansion, has been observed in several ornamental species subjected to deficit irrigation, including *Callistemon citrinus*, *Pittosporum*, *Viburnum*, and *Cistus* species [53,55,61,62]. In our study, partial compensation for evapotranspiration losses led to a marked decrease in total leaf area, primarily due to the restricted expansion of individual leaves. This response is considered an avoidance mechanism that limits transpirational water loss through stomatal closure, thereby reducing whole-plant carbon assimilation and growth [63].

Drought stress responses are inherently complex and energy-intensive, involving the activation of interconnected physiological and biochemical processes that vary according to genotype, plant developmental stage, and the intensity and duration of the stress [36]. Consequently, plant performance under drought is often evaluated using indirect indicators, such as photosynthetic activity, gas exchange, and metabolite accumulation. However, unlike many ornamental species that experience significant reductions in biomass production and ornamental quality under drought conditions, *C. amboinicus* maintains relatively stable growth and physiological activity. This suggests the presence of coordinated drought-avoidance mechanisms involving morphological, physiological, and biochemical adjustments. In this context, Cuban oregano behaves as a facultative CAM species under water-limited conditions [64], a trait that contributes to improved water-use efficiency and enhanced tolerance to prolonged water deficit [65].

In our experiment, increasing water stress led to a reduction in CO_2_ assimilation and net photosynthesis, a response linked to increased resistance at both the stomatal and mesophyll levels [66]. Rapid stomatal closure is an early response to water scarcity, resulting in decreases in stomatal conductance, transpiration rate, and net photosynthetic rate [67,68]. This regulation of stomata is a key control mechanism in maintaining the plant’s water balance, although it results in a reduction in carbon assimilation. Despite these declines, Cuban oregano demonstrated the ability to partially acclimate through coordinated physiological adjustments. While stomatal closure serves as a defence mechanism to minimise water loss during drought, it can also diminish photosynthetic efficiency.

Metabolic responses further substantiated the mechanisms of drought tolerance. One objective of this study was to assess the accumulation of protective compounds under water stress conditions. Reducing sugars tend to accumulate in the roots of stressed plants, while sugar concentrations in the leaves decline. This pattern reflects reduced photosynthetic activity during drought, which limits carbohydrate biosynthesis, particularly under severe stress [69]. The relatively minor reduction observed in this study supports the classification of *C. amboinicus* as a drought-tolerant species.

Carotenoids play a vital role in photoprotection by dissipating excess energy and scavenging reactive oxygen species (ROS). The observed increase in carotenoid content suggests an enhanced photoprotective capacity, indicating the activation of antioxidant defence mechanisms that protect the photosynthetic apparatus from oxidative damage under stress conditions. Similarly, the reduction in nitrate concentration likely reflects a combination of restricted root uptake, reduced xylem transport, and increased nitrogen utilisation for the biosynthesis of osmolytes such as proline.

Soluble sugars and other compatible osmolytes accumulate under drought stress, aiding osmotic adjustment, membrane stabilisation, and ROS scavenging. This osmotic adjustment mechanism is crucial for maintaining cell turgor and ensuring enzyme functionality during dehydration. The ability of WE at 1 mL L^−1^ to maintain sugar levels comparable to those of well-watered plants indicates a protective effect mediated by the biostimulant. Osmolyte accumulation, particularly proline, is a key indicator of drought tolerance, as proline reduces cellular osmotic potential, stabilises proteins and membranes, and alleviates oxidative stress [70]. Among the osmolytes analysed, proline showed preferential accumulation in leaves, especially under the 50% WC + WE 5 mL L^−1^ treatment, suggesting a role in cellular protection during water deficit conditions.

Chlorophyll content, measured using the SPAD index, consistently decreased under water deficit conditions compared with fully irrigated plants. Water stress impairs chlorophyll biosynthesis and accelerates its degradation, resulting in lower pigment levels and diminished leaf greenness. Nevertheless, the continuation of functional photosynthesis despite pigment loss indicates the activation of protective mechanisms that preserve the integrity of the photosystem. Maintaining higher SPAD values has been linked to increased drought tolerance, while reduced chlorophyll content may adversely affect the ornamental value of plants [71].

Overall, changes in photosynthesis, gas exchange, and metabolite accumulation confirmed that photosynthetic parameters and selected biochemical traits are reliable indicators of plant adaptation to drought. In this context, the application of a biostimulant derived from *Crithmum maritimum* partially and positively modified the plant’s responses to water stress. Plant-derived biostimulants represent a sustainable strategy for enhancing stress tolerance, improving growth, and supporting low-input agricultural systems [72]. The biostimulant obtained through alcoholic extraction exhibited a markedly higher content of total soluble solids and total polyphenols. These compounds are likely responsible for enhancing antioxidant capacity, thereby mitigating oxidative stress under drought conditions; conversely, the aqueous extract has a different effect. Alcoholic extraction also preserved a modest amount of vitamin C, a compound for which *Crithmum maritimum* is known to be rich. The vitamin C was found almost entirely in its oxidised form, whereas it was completely absent in the aqueous macerate. Conversely, the latter retained a higher amount of proteins and likely peptides, which are known to precipitate in the presence of alcohol.

Consistent with previous studies on plant-derived biostimulants, including extracts from moringa, liquorice, garlic, and protein hydrolysates, our findings demonstrated that the efficacy of biostimulants strongly depends on both the dosage and the extraction method. We found that *C. amboinicus* responds to water deficit through multiple adaptive mechanisms, such as biomass reallocation towards the roots, stomatal regulation, osmotic adjustment, and enhanced antioxidant protection. Furthermore, the positive effects observed following the application of *C. maritimum* extracts suggest that plant-derived biostimulants may improve plant performance under water-limited conditions [73]. These results support the potential use of *C. amboinicus* as a low-input ornamental species and indicate that natural biostimulants could offer a sustainable strategy to mitigate the effects of drought stress in ornamental horticulture.

## 4. Materials and Methods

### 4.1. Plant Material and Experimental Conditions

The trial was conducted in a greenhouse at the Department of Agriculture, Food and Environment (Di3A) in Catania, Italy (37°41′ N, 15°11′ E, 89 metres above sea level) from 7 April to 6 June 2025, using Cuban oregano (*Coleus amboinicus* Lour.). On 5 November 2024, top cuttings from plants in the collection of the Vegetable and Floriculture Section of the Department of Agriculture, Food and Environment of Catania University were rooted in a cold greenhouse in 2-litre containers (Figure S1).

**Figure 11:**
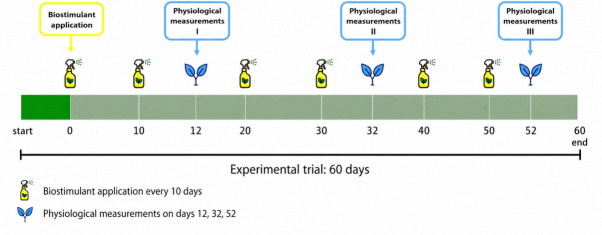
A timeline of the experimental trial and the highlighted treatment application and physiological measurements.

The growing medium used was supplied by Vigorplant Italia srl (Fombio, LO, Italy) and consisted of acidic sphagnum peat and pumice. To prevent water stagnation, a light layer of pH-controlled expanded clay (Vigorplant, Fombio, LO, Italy) was placed at the bottom of each container. On 21 January 2025, the plants were topped and transferred into 17 cm diameter, 3-litre containers filled with the same substrate described above. No fertilisation was applied during the trial period. Temperatures in February and March 2025 were particularly mild, and there were several overcast days. Throughout the experimental period, the mean air temperature was approximately 24 °C, although maximum daily temperatures frequently exceeded 40 °C (Figure S2). Consequently, the plants were regularly irrigated at field capacity (100%) until 7 April 2025, which marked the start date of the trial (Figure 11). Each pot was uniquely identified by a tag; control plants were irrigated by replacing 100% of the water lost through evapotranspiration (100% WC), while the 50% WC plants received 50% replacement.

The plants at 50% WC were treated every 10 days using a pressure pump with a maximum capacity of two litres (Matabi, GreenCity2 model, Goizper S. Coop. Antigua, Antzuola, Spain). Plants were sprayed with the various treatments until runoff; control plants were sprayed with distilled water.

#### 4.1.1. Water Extract (WE)

Fresh *C. maritimum* leaves were blended and macerated in distilled water at a 1:2 (*w*/*v*) ratio for 21 days. The resulting extract served as the stock solution for foliar applications. Plants were sprayed to runoff with WE at concentrations of 1, 5, and 10 mL L^−1^ (Table 3).

#### 4.1.2. Alcoholic Extract (AE)

Fresh *C. maritimum* leaves were extracted using a mixture of 96% ethanol and 4% methanol. The alcoholic extracts were concentrated under reduced pressure using a rotary evaporator (BUCHI Rotavapor R-100, BUCHI Italia s.r.l., Cornaredo, Italy) and then resuspended in distilled water to achieve the same biomass-to-solvent ratio (*w*/*v*) as the aqueous extract (Table 3).

Irrigation treatments were managed using the gravimetric method [74]. Throughout the experimental period, differences in pot weight (weight after irrigation at drainage cessation minus weight before irrigation) were calculated, and irrigation was carried out three times per week.

The air temperature and relative humidity inside the greenhouse were recorded using a Mini TH data logger (IP30 protection class, equipped with a protective shell and an integrated USB port; Tecnafood, Bomporto, MO, Italy). During the trial, the mean temperature was 23.2 °C, reaching a maximum of 52 °C; notably, in the final phase, maximum temperatures often exceeded 40 °C. The mean relative humidity was 50.2%, with peaks of 88% and a minimum of 8%; in the last phase of the trial, RH frequently fell below 60%.

### 4.2. Biostimulants Chemical Characterisation

Biostimulants obtained from aqueous maceration and alcoholic extraction of *C. maritimum* leaves were characterised in terms of soluble solids content, pH, titratable acidity, total phenolics, total proteins, and vitamin C. Analyses were conducted on aliquots from the same bulk sample, each independently centrifuged at 13,500 rpm for 10 min. Results are presented as the mean of two analytical replicates.

#### 4.2.1. Total Soluble Solids, pH, and Titratable Acidity

Total soluble solids (TSS, expressed as °Brix), pH and total titratable acidity (TTA, expressed as mEq L^−1^) were measured according to Fibiani et al. [75] (Table 4).

#### 4.2.2. Total Polyphenol Content

Total polyphenol content (TPC) was determined using the Folin–Ciocalteu (F–C) assay following the method of Bulgari et al. [76], with some modifications. Fifty microlitres of the sample were diluted with 2 mL of distilled water, after which 62 μL of F–C reagent was added. After standing at room temperature for 5 min, 370 μL of 20% Na_2_CO_3_ solution was introduced. The mixture was briefly vortexed and then incubated in the dark for 2 h. Absorbance was measured at 740 nm using a Jasco UV–Vis spectrophotometer (V630, Jasco-Europe, Lecco, Italy). Calibration curves were prepared using gallic acid standards, and results were expressed as mg L^−1^ gallic acid equivalents (GAE) (Table 4).

#### 4.2.3. Protein Content

Protein content (PC), encompassing proteins and possibly medium- to high-molecular-weight peptides, was measured using the Bradford assay with bovine serum albumin (BSA) as the standard [77]. In brief, 100 μL of the sample was mixed with 900 μL of Bradford reagent, gently inverted, incubated for 5 min, and the absorbance was read at 595 nm. Results are expressed as mg L^−1^ BSA equivalents (Table 4).

#### 4.2.4. Vitamin C Determination

Vitamin C, determined as the combined total of ascorbic acid (AA) and dehydroascorbic acid (DHAA), was quantified using RP-UV-HPLC following the method of Fibiani et al. [75]. The results are presented as mg L^−1^ (Table 4).

### 4.3. Biomass and Leaf Area

Biomass data were collected at the start of the trial by destructively sampling four plants to determine the fresh and dry biomass of the various plant organs. Roots were separated from the substrate by carefully washing under running water, while shoots were divided into stems and leaves.

Dry biomass was obtained by oven-drying samples at 70 °C in a ventilated oven until a constant weight was reached. Leaf area and the number of leaves per plant were also determined.

The number of leaves and their area were measured by placing leaves on a white background, covering them with a transparent glass plate to keep them fully extended, and then photographing them. The images were processed using ImageJ software (https://imagej.net/ij/, accessed on 23 June 2025).

### 4.4. SPAD Index, Gas Exchange, and Chlorophyll a Fluorescence

Physiological measurements were taken two days after the biostimulant treatments. To provide a clearer understanding of the results, the three most significant findings (Measurements I, II, and III) have been included in the manuscript.

Chlorophyll content was indirectly estimated using the SPAD index, measured on ten leaves from four plants per treatment with a portable chlorophyll metre (SPAD-502, Minolta Camera Co., Osaka, Japan).

Gas exchange was measured on six plants per treatment (two plants per replicate, with two leaves per plant) using a CO_2_/H_2_O Infrared Gas Analyzer (LCi, ADC Bioscientific Ltd., Hoddesdon, UK). Measurements were taken in the morning under clear sky conditions between 09:00 and 13:00. Net photosynthesis (A_n_), stomatal conductance (g_s_), and transpiration rate (E) were recorded.

Simultaneously, the PSII quantum yield was measured using a fluorimeter (OS1-FL, Opti-Sciences Corporation, Tyngsboro, MA, USA). Leaves were dark-adapted for 20 min using cuvette clips (Opti-Sciences) prior to measurements. Chlorophyll *a* fluorescence was expressed as Fv/Fm, where Fm represents the maximal fluorescence of dark-adapted leaves and Fv denotes variable fluorescence.

### 4.5. Nitrate and Reducing Sugar Content, and Osmolyte Concentration

The nitrate content in plant tissues was determined according to the method of Cataldo et al. [78]. Dried samples (100 mg) were ground, placed in 15 mL Falcon tubes, and extracted with 3 mL of distilled water. The tubes were shaken for 2 h and then centrifuged at 4000 rpm for 15 min. A 20 µL aliquot of supernatant was transferred to a 5 mL Eppendorf tube, mixed with 80 µL of salicylic-sulfuric acid (5% *w*/*v*), and 3 mL of NaOH (1.5 N). After cooling to room temperature, the absorbance was measured at 410 nm using a spectrophotometer (Infinite M Nano, Tecan Trading AG, Männedorf, Switzerland). Calibration curves were prepared using KNO_3_ standard solutions at concentrations of 0, 2.5, 5, 7.5, and 10 mM.

Reducing sugars were determined using the DNS (3,5-dinitrosalicylic acid) method. Dried samples (0.1 g) were ground and placed in 15 mL Falcon tubes with 3 mL of distilled water, shaken for 2 h and centrifuged at 10 °C for 15 min at 4000 rpm. A 200 µL aliquot of the supernatant was transferred into an Eppendorf tube, mixed with 0.2 mL of the DNS reagent, vortexed, and incubated in a dry bath thermostat (ThermoStat plus, Eppendorf, Hamburg, Germany) at 99 °C for 5 min. The completion of the reaction was confirmed by a distinct colour change upon reaching the target temperature. After adding 1.5 mL of distilled water and cooling to room temperature, absorbance was measured at 530 nm using the Infinite M Nano spectrophotometer (Tecan). Calibration was performed using a glucose standard solution at concentrations of 0, 1, 2, 3, and 4 mM.

The osmolyte concentration was measured in a water extract with a freezing point osmometer (Osmomat 030, Gonotec, Logan, UT, USA). This method relies on freezing point depression, which is directly proportional to the concentration of osmotically active particles in the solution. The instrument was calibrated before use with distilled water (zero point) and a standard solution (upper reference point). Results were expressed in osmol/kg.

### 4.6. Chlorophyll and Carotenoid Concentration

Chlorophyll *a* + *b* and carotenoids were extracted from leaves using 99.9% (*v*/*v*) methanol. Three leaf disc samples (30 mg each), obtained with a 7 mm diameter cork borer, were kept in a dark room at 4 °C for 24 h in 5 mL tubes containing 4 mL of methanol. The pigment content was determined colorimetrically, with absorbance readings taken at 665 nm and 652 nm for chlorophylls and 470 nm for total carotenoids, using a spectrophotometer (Infinite 200 Pro, Tecan Trading AG, Männedorf, Switzerland). Pigment levels were calculated according to Lichtenthaler’s formula [79] and expressed on a dry weight basis.

### 4.7. Anthocyanin Concentration

Anthocyanins were extracted using 3 mL of methanol acidified with 1% HCl. Leaf disc samples (30 mg), obtained with a 7 mm diameter cork borer, were kept in a dark room at 4 °C for 24 h in 5 mL tubes filled with acidified methanol. Absorbance readings for anthocyanin were then measured at 535 nm using a spectrophotometer (Infinite 200 Pro, Tecan Trading AG, Männedorf, Switzerland). Concentrations were expressed as cyanidin-3-glucoside equivalents, calculated using the molar extinction coefficient in acidified methanol (ε FW Anthocyanin) of 34,300 L mol^−1^ cm^−1^ [80].

### 4.8. Amino Acid Profile with HPLC-MS/MS

Amino acid profiling was conducted using targeted quantitative analysis, following the protocol established by Fidalgo-Illesca et al. [81], with slight modifications. Metabolites were extracted using a solvent-based method. Frozen samples from each treatment were homogenised with liquid nitrogen, avoiding exposure to light to ensure consistent extraction. Methanol/milliQ water (80/20, *v*/*v*) was used as the extraction solvent at a ratio of 1:10 (*w*/*v*), with 0.500 g of plant tissue combined with 5 mL of solvent in a 15 mL tube. The samples were shaken overnight in the dark at 4 °C. Subsequently, the extracts were centrifuged for 15 min at 4000 rpm. The supernatants were collected and filtered through a 0.45 μm pore size membrane before identification and quantification of the target compounds by LC-MS/MS. This was performed using a Sciex 5500 QTrap+ mass spectrometer (AB Sciex LLC, Framingham, MA, USA), equipped with a Turbo V ion-spray source and coupled to a custom-made ExionLC AC System by Shimadzu (Shimadzu Corporation, Kyoto, Japan), which includes an ExionLC Controller, ExionLC Degasser, two ExionLC AC Pumps, and an ExionLC AC Autosampler.

The UHPLC chromatographic separation for the targeted quantification of amino acids was carried out using a Biphenyl HPLC column (100 × 2.1 mm, 2.6 μm). Elution was performed with acetonitrile/water (*v*/*v*, 15/85) containing 0.1% (*v*/*v*) formic acid as the mobile phase, at a flow rate of 400 μL min^−1^. The injection volume was 20 μL, and the column oven temperature was maintained at 40 °C. MS/MS analysis was conducted in electrospray negative ion mode, using nitrogen as the collision gas.

Calibration curves for amino acids were prepared using the Amino Acids Mix Solution standard (Supelco^®^, Sigma, Merck KGaA, Darmstadt, Germany) at concentrations ranging from 0.01 to 2.56 nmol mL^−1^.

### 4.9. Statistical Analysis

Data were analysed using one-way ANOVA, with biostimulant treatments as factors, employing CoStat 6.311 (CoHort Software, Monterey, CA, USA). Mean differences were assessed using Tukey’s post hoc test (*p* < 0.05). Physiological data are presented as means ± SE (n = 3).

## 5. Conclusions

The present study demonstrated that extracts from *Crithmum maritimum* can effectively alleviate the adverse effects of water deficit in *Coleus amboinicus*. The biostimulant response was clearly influenced by both the extraction method and dosage. In particular, alcoholic extracts proved more effective than aqueous extracts, significantly enhancing biomass accumulation, leaf development, and physiological performance under drought conditions. The most effective treatment (AE at 2.5 mL L^−1^) was able to partially restore growth parameters to levels comparable with those of well-watered plants, confirming its strong potential as a biostimulant under stress conditions.

*C. maritimum* is recognised for its medicinal properties, yet its potential as a plant biostimulant remains largely unexplored. Further research is required to determine optimal extraction methods, appropriate dosages, and timing of application, as well as to assess its effectiveness in comparison with other plant-based and commercial biostimulants. Given that plant responses vary between species, future studies should investigate the effects of *C. maritimum* extracts on a wider range of ornamental species and genotypes. Understanding the underlying mechanisms of its action is essential for developing broadly applicable and efficient biostimulant strategies to enhance plant resilience, particularly under water-limited conditions.

## Figures and Tables

**Table 1:** Effects of different biostimulant treatments at the end of the trial on total, epigeous, leaf, stem and root dry biomass; root/shoot ratio (R/S).

Treatments	Total Dry Biomass (g plant^−1^)	Epigeous Dry Biomass (g plant^−1^)	Leaf Dry Biomass (g plant^−1^)	Stem Dry Biomass (g plant^−1^)	Root Dry Biomass (g plant^−1^)	R/S Ratio
100% WC	40.2 ± 1.3 a	34.8 ± 1.3 a	18.0 ± 0.5 a	16.7 ± 0.8 a	5.45 ± 0.1 ab	0.16 ± 0.0 b
50% WC	21.0 ± 1.9 d	17.1 ± 1.5 d	9.46 ± 0.6 de	7.65 ± 0.8 d	3.87 ± 0.5 bc	0.22 ± 0.01 a
50% WC-AE 0.25 mL L^−1^	24.1 ± 0.1 cd	20.4 ± 0.3 cd	10.8 ± 0.0 cd	9.60 ± 0.3 cd	3.63 ± 0.2 c	0.18 ± 0.01 ab
50% WC-AE 1.25 mL L^−1^	23.9 ± 0.8 cd	19.7 ± 0.4 cd	10.7 ± 0.2 cd	9.00 ± 0.3 cd	4.19 ± 0.4 bc	0.21 ± 0.02 ab
50% WC-AE 2.5 mL L^−1^	33.7 ± 0.1 b	27.8 ± 0.6 b	14.4 ± 0.2 b	13.4 ± 0.3 b	5.90 ± 0.5 a	0.21 ± 0.02 ab
50% WC-WE 1 mL L^−1^	21.3 ± 0.2 d	17.8 ± 0.3 d	8.56 ± 0.7 e	9.23 ± 0.4 cd	3.55 ± 0.1 c	0.20 ± 0.01 ab
50% WC-WE 5 mL L^−1^	26.4 ± 0.7 c	22.0 ± 0.3 c	12.0 ± 0.1 c	10.0 ± 0.1 cd	4.43 ± 0.4 abc	0.20 ± 0.02 ab
50% WC-WE 10 mL L^−1^	27.6 ± 0.8 c	22.9 ± 0.7 c	12.7 ± 0.4 bc	10.2 ± 0.3 c	4.72 ± 0.2 abc	0.21 ± 0.02 ab
Significance	***	***	***	***	***	*

Abbreviations: 100% WC = received 100% of water considering the evapotranspiration rate performed using the gravimetric method and were foliar spraying to runoff with distilled water; 50% WC = received 50% of water considering the evapotranspiration rate performed using the gravimetric method and were foliar spraying to runoff with distilled water; AE = biostimulant extraction from leaves of *C. maritimum* by ethanol (96%) and methanol (4%); WE = biostimulant obtained from water maceration of leaves of *C. maritimum*. Data are means ± standard error (n = 3). Data followed by a different letter were significantly different according to Tukey’s test at *p* < 0.05; * *p* < 0.05; *** *p* < 0.001.

**Table 2:** Effects of different biostimulant treatments at the end of the trial on leaf number, total leaf area, unit leaf area, and specific leaf area (SLA).

Treatments	Leaf Number (n)	Total Leaf Area (cm^2^ plant^−1^)	Unit Leaf Area (cm^2^)	SLA
100% WC	935.0 ± 57.2 a	2617.1 ± 80.1 a	2.82 ± 0.19 abc	145.4 ± 5.5 bc
50% WC	622.3 ± 8.20 b	1115.1 ± 66.7 e	1.79 ± 0.08 d	118.6 ± 8.6 d
50% WC-AE 0.25 mL L^−1^	735.1 ± 38.8 b	1780.4 ± 13.3 bc	2.44 ± 0.11 bcd	164.3 ± 1.0 ab
50% WC-AE 1.25 mL L^−1^	657.2 ± 34.3 b	1765.1 ± 9.10 bc	2.71 ± 0.16 abc	165.1 ± 3.3 ab
50% WC-AE 2.5 mL L^−1^	769.0 ± 9.50 b	2584.5 ± 20.8 a	3.36 ± 0.01 a	180.2 ± 1.2 a
50% WC-WE 1 mL L^−1^	458.8 ± 35.6 c	1281.7 ± 121.6 de	2.78 ± 0.05 abc	149.5 ± 1.4 b
50% WC-WE 5 mL L^−1^	680.4 ± 27.6 b	1949.3 ± 53.6 b	2.89 ± 0.20 ab	163.2 ± 6.5 ab
50% WC-WE 10 mL L^−1^	718.5 ± 17.0 b	1552.0 ± 110.9 cd	2.17 ± 0.21 cd	121.7 ± 5.3 cd
Significance	***	***	***	***

Abbreviations: 100% WC = received 100% of water considering the evapotranspiration rate performed using the gravimetric method and were foliar spraying to runoff with distilled water; 50% WC = received 50% of water considering the evapotranspiration rate performed using the gravimetric method and were foliar spraying to runoff with distilled water; AE = biostimulant extraction from leaves of *C. maritimum* by ethanol (96%) and methanol (4%); WE = biostimulant obtained from water maceration of leaves of *C. maritimum*. Data are means ± standard error (n = 3). Data followed by a different letter were significantly different according to Tukey’s test at *p* < 0.05; ***: *p* < 0.001.

**Table 3:** Foliar treatments applied to plants grown under 50% water capacity.

Biostimulant	Extraction Method	Application Rate (mL L^−1^)
50% WC-WE 1 mL L^−1^	Aqueous maceration of *Crithmum maritimum* leaves	1
50% WC-WE 5 mL L^−1^	Aqueous maceration of *C. maritimum* leaves	5
50% WC-WE 10 mL L^−1^	Aqueous maceration of *C. maritimum* leaves	10
50% WC-AE 0.25 mL L^−1^	Alcoholic extraction of *C. maritimum* leaves	0.25
50% WC-AE 1.25 mL L^−1^	Alcoholic extraction of *C. maritimum* leaves	1.25
50% WC-AE 2.5 mL L^−1^	Alcoholic extraction of *C. maritimum* leaves	2.5

**Table 4:** Chemical characterisation of biostimulants obtained from *Crithmum maritimum* leaves.

Biostimulant	TSS ^a^ (°Brix)	pH	TTA ^b^ (mEq L^−1^)	TPC ^c^ (mg L^−1^ GAE)	PC ^d^ (mg L^−1^ BSA)	Vitamin C	DHAA ^e^ (%)
WE (aqueous maceration)	1.84	5.04	6.6	418	48	n.d.	n.d.
AE (alcoholic extraction)	4.09	4.86	6.2	1351	14	118	89

^a^ total soluble solids; ^b^ total titratable acidity; ^c^ total polyphenol content (gallic acid equivalents); ^d^ protein content (bovine serum albumin equivalents); ^e^ dehydroascorbic acid. n.d. = not detected.

## Data Availability

The raw data supporting the conclusions of this article will be made available by the authors on request.

## Supplementary Materials

The following supporting information can be downloaded at: https://www.mdpi.com/article/10.3390/plants15142107/s1, Table S1: Amino Acid Profiles with HPLC-MS/MS Quantification. Figure S1: Plants subjected to the different water and biostimulant treatments at the end of the experimental trial. Figure S2: Temperature (°C) and Relative Humidity (%) during the experimental period.
