# Supplementary material for: Influence of Dose and Extraction Method of Biostimulants on Drought Stress Tolerance in Coleus amboinicus Lour. Plants
Source: Plants (Basel). 2026 Jul 8;15(14):2107. doi: 10.3390/plants15142107 (PMC13414684; doi:10.3390/plants15142107)
Supplement: Supplementary file 1 [file plants-15-02107-s001.zip › plants-4388139-supplementary.pdf]

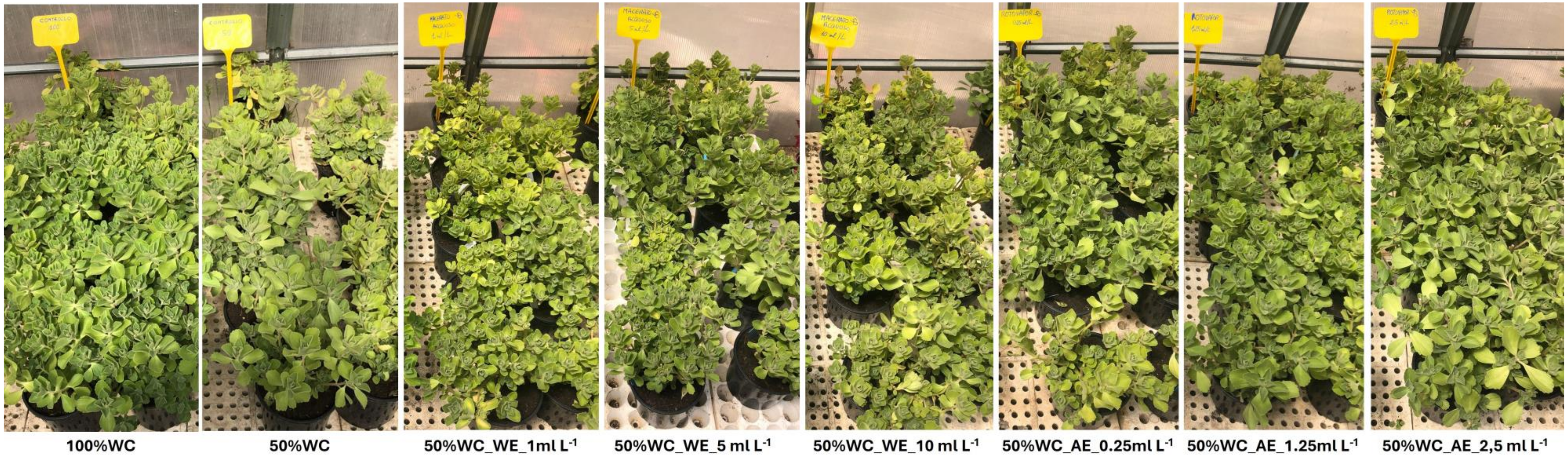

Figure S1. Plants subjected at the different water and biostimulant treatments at the end of the experimental trial.

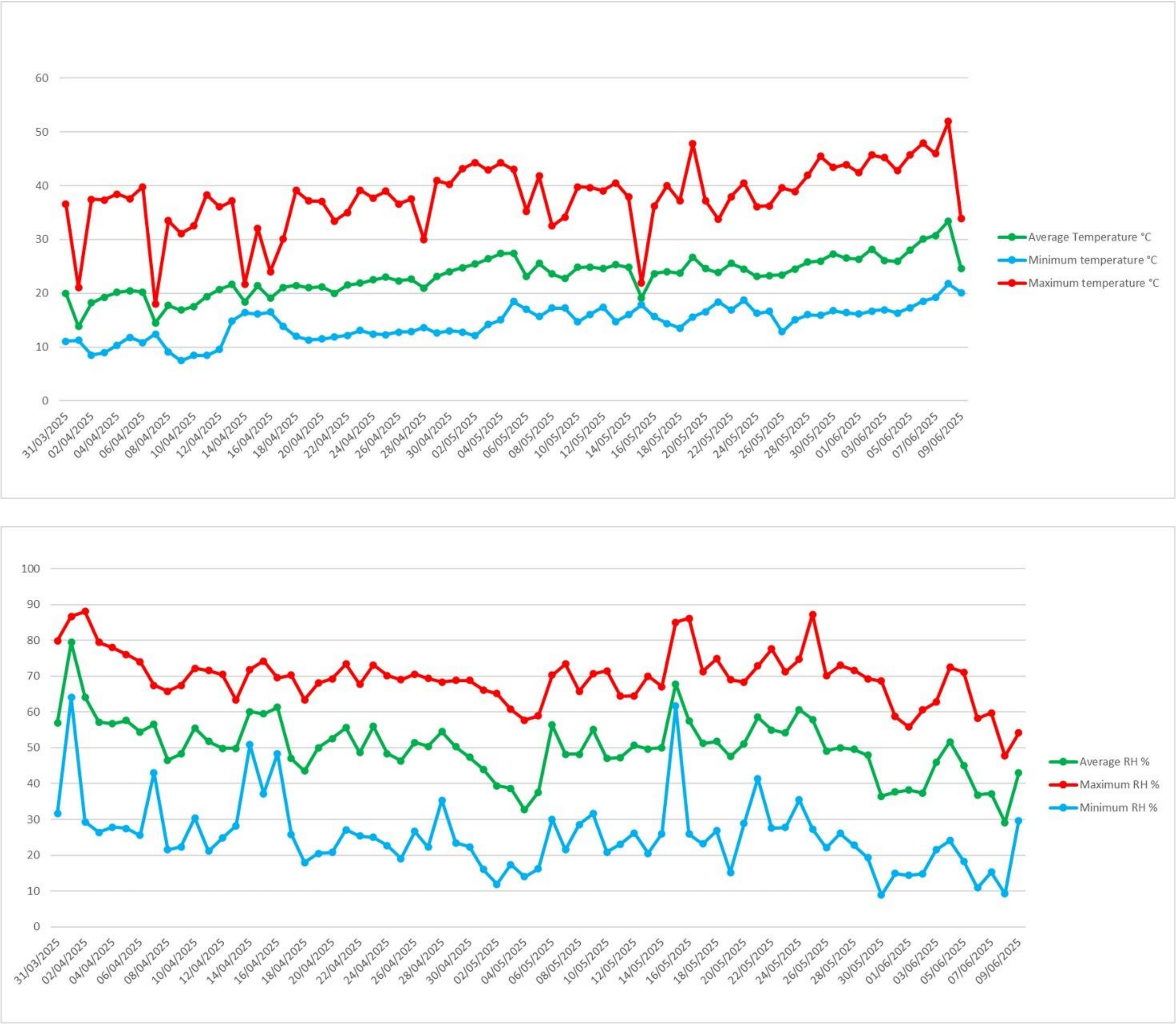

Figure S2. Temperature (°C) and Relative Humidity (%) during the experimental period.

TableS1. Amino Acid Profiles with HPLC-MS/MS Quantification.

Treatment	Glycine		Alanine		Serine		Valine		Lysine		Methionine	
	Mean	SE	Mean	SE	Mean	SE	Mean	SE	Mean	SE	Mean	SE
**100%WC**	0.003295	0.000025	0.013055	0.008444	0.009565	0.004293	0.012163	0.001552	0.000122	0.000010	0.000382	0.000091
**50%WC**	0.008854	0.004367	0.003507	0.001368	0.010807	0.008388	0.043389	0.003158	0.079819	0.010278	0.000679	0.000136
**50%WC_AE_0.25ml L^{-1}**	0.004839	0.002377	0.029906	0.013900	0.007787	0.006438	0.020656	0.000463	0.059712	0.031021	0.000743	0.000075
**50%WC_AE_1.25ml L^{-1}**	0.008510	0.001144	0.022573	0.020406	0.012781	0.011570	0.041426	0.003909	0.018253	0.018081	0.000580	0.000157
**50%WC_AE_2.5ml L^{-1}**	0.016867	0.002048	0.026582	0.011097	0.024254	0.000564	0.029691	0.001682	0.035181	0.034973	0.000689	0.000137
**50%WC_WE_1ml L^{-1}**	0.004488	0.001396	0.021725	0.017596	0.010680	0.009314	0.028250	0.004153	0.015037	0.014632	0.000614	0.000096
**50%WC_WE_5ml L^{-1}**	0.041386	0.015191	0.024550	0.019198	0.043547	0.005262	0.064962	0.011997	0.000156	0.000038	0.001405	0.000418
**50%WC_WE_10ml L^{-1}**	0.004072	0.000281	0.012522	0.008137	0.009777	0.003525	0.014699	0.002107	0.020979	0.011651	0.000658	0.000097

Treatment	Histidine		Phenylalanine		Arginine		Tyrosine		Cysteine	
	Mean	SE	Mean	SE	Mean	SE	Mean	SE	Mean	SE
**100%WC**	0.003282	0.000468	0.011805	0.001739	0.010570	0.001901	0.000993	0.000060	0.000548	0.000250
**50%WC**	0.005642	0.001296	0.060590	0.001847	0.103467	0.012121	0.026241	0.004227	0.001521	0.000525
**50%WC_AE_0.25ml L^{-1}**	0.003938	0.000472	0.018936	0.001418	0.033474	0.002949	0.001124	0.000091	0.001066	0.000368
**50%WC_AE_1.25ml L^{-1}**	0.003651	0.000886	0.065393	0.006662	0.063632	0.015453	0.000506	0.000091	0.000789	0.000599
**50%WC_AE_2.5ml L^{-1}**	0.003211	0.000073	0.030286	0.002097	0.056449	0.006625	0.000955	0.000057	0.001100	0.000464
**50%WC_WE_1ml L^{-1}**	0.004021	0.000403	0.035841	0.005966	0.029119	0.003644	0.000548	0.000104	0.000507	0.000356
**50%WC_WE_5ml L^{-1}**	0.004112	0.000273	0.073083	0.009477	0.104474	0.008277	0.000615	0.000056	0.000960	0.000476
**50%WC_WE_10ml L^{-1}**	0.005826	0.001952	0.012959	0.000740	0.019914	0.003557	0.001216	0.000073	0.000692	0.000594
